# Production of mannosylglycerate in *Saccharomyces cerevisiae* by metabolic engineering and bioprocess optimization

**DOI:** 10.1186/s12934-018-1023-7

**Published:** 2018-11-16

**Authors:** Cristiana Faria, Nuno Borges, Isabel Rocha, Helena Santos

**Affiliations:** 1 Centre of Biological Engineering, University of Minho, Braga, Portugal; 2 Instituto de Tecnologia Química e Biológica António Xavier, Universidade Nova de Lisboa (ITQB NOVA), Oeiras, Portugal

**Keywords:** Mannosylglycerate, Compatible solute, GDP-mannose, Yeast cell factory, Metabolic engineering, Chemostat cultivation

## Abstract

**Background:**

Mannosylglycerate (MG) is one of the most widespread compatible solutes among marine microorganisms adapted to hot environments. This ionic solute holds excellent ability to protect proteins against thermal denaturation, hence a large number of biotechnological and clinical applications have been put forward. However, the current prohibitive production costs impose severe constraints towards large-scale applications. All known microbial producers synthesize MG from GDP-mannose and 3-phosphoglycerate via a two-step pathway in which mannosyl-3-phosphoglycerate is the intermediate metabolite. In an early work, this pathway was expressed in *Saccharomyces cerevisiae* with the goal to confirm gene function (Empadinhas et al. in *J Bacteriol* 186:4075–4084, 2004), but the level of MG accumulation was low. Therefore, in view of the potential biotechnological value of this compound, we decided to invest further effort to convert *S. cerevisiae* into an efficient cell factory for MG production.

**Results:**

To drive MG production, the pathway for the synthesis of GDP-mannose, one of the MG biosynthetic precursors, was overexpressed in *S. cerevisiae* along with the MG biosynthetic pathway. MG production was evaluated under different cultivation modes, i.e., flask bottle, batch, and continuous mode with different dilution rates. The genes encoding mannose-6-phosphate isomerase (*PMI40*) and GDP-mannose pyrophosphorylase (*PSA1*) were introduced into strain MG01, hosting a plasmid encoding the MG biosynthetic machinery. The resulting engineered strain (MG02) showed around a twofold increase in the activity of *PMI40* and *PSA1* in comparison to the wild-type. In batch mode, strain MG02 accumulated 15.86 mg_MG_ g_DCW_^−1^, representing a 2.2-fold increase relative to the reference strain (MG01). In continuous culture, at a dilution rate of 0.15 h^−1^, there was a 1.5-fold improvement in productivity.

**Conclusion:**

In the present study, the yield and productivity of MG were increased by overexpression of the GDP-mannose pathway and optimization of the mode of cultivation. A maximum of 15.86 mg_MG_ g_DCW_^−1^ was achieved in batch cultivation and maximal productivity of 1.79 mg_MG_ g_DCW_^−1^ h^−1^ in continuous mode. Additionally, a positive correlation between MG productivity and growth rate/dilution rate was established, although this correlation is not observed for MG yield.

**Electronic supplementary material:**

The online version of this article (10.1186/s12934-018-1023-7) contains supplementary material, which is available to authorized users.

## Background

Enzymes and other proteins are used in a myriad of applications such as clinical and analytical tests, research, therapeutics, vaccines, food processing, textile industry, cleaning, and biofuel production. In every case, the preservation of the native structure under working conditions is a prerequisite for efficacy. While some proteins are remarkably robust and can stand harsh conditions or repeated work cycles, others require the presence of extrinsic stabilizers, or chemical chaperones, to prevent unfolding and/or assist refolding. Osmolytes, like glycerol and trehalose, which accumulate inside the cell to counterbalance the external osmotic pressure, are well known protein protectors [1, 2].

The discovery of marine hyperthermophilic organisms in the early 1980s uncovered an untapped source of new osmolytes [3]. Microorganisms adapted to those extreme environments accumulate exquisite organic solutes, usually bearing a negative charge, which are absent from mesophilic prokaryotes. Importantly, these ionic solutes accumulate not only in response to elevated osmotic pressure, but also in response to supraoptimal growth temperature, suggesting a potential role in thermoprotection of cell components in vivo [4].

Mannosylglycerate and di-*myo*-inositol-phosphate are the most widespread solutes among marine hyperthermophiles [2–5]. In vitro studies have shown that these compounds are better protein stabilizers than trehalose, glycerol or any other known neutral solutes [6–11]. In particular, MG showed superior performance in a wide range of application tests [6–12]. For example, 0.5 M of MG resulted in a 7.1 °C increase in the melting temperature of the model protein, staphylococcal nuclease, while the same concentration of glycerol or mannosylglyceramide produced increments of < 1 °C and 2.4 °C, respectively [13]. Importantly, we showed that MG was able to inhibit formation of α-synuclein inclusions in the cytoplasm of yeast cells, a hint of its potential application in drug development against protein misfolding diseases [13, 14].

All known native producers, Archaea and Bacteria, are able to synthesize MG via a two-step pathway: the enzyme mannosyl-3-phosphoglycerate synthase catalyzes the reaction of GDP-mannose and 3-phosphoglycerate into mannosyl-3-phosphoglycerate, which is subsequently dephosphorylated by mannosyl-3-phosphoglycerate phosphatase to give MG [15, 16]. Empadinhas et al. [17] noticed that the genome of the mesophilic bacterium *Dehalococcoides mccartyi* (formerly *D. ethenogenes*), comprised a gene encoding two domains with high sequence homology to known mannosyl-3-phosphoglycerate synthase and mannosyl-3-phosphoglycerate phosphatase. The same authors confirmed the functionality of this gene, designated *mgsD*, by cloning and expression in *Saccharomyces cerevisiae*, which thereby accumulated intracellular MG up to 7.5 mg g^−1^ cell dry weight [17].

Capitalizing on these results, we deemed it interesting to invest further efforts to develop *S. cerevisiae* into a cell factory for MG production. Currently, MG is produced by bitop AG (Witten, Germany) through fermentation of a natural producer. However, this process has very high production costs, preventing the utilization of this compound at an industrial scale or even extensive testing to explore new application areas. We selected *S. cerevisiae* as a host organism for the engineering strategy since it is a robust industrial workhorse used for the production of a wide range of compounds, such as sesquiterpenes, bioethanol, artemisinic acid, succinic acid and vanillin [18–22].

**Fig. 1:**
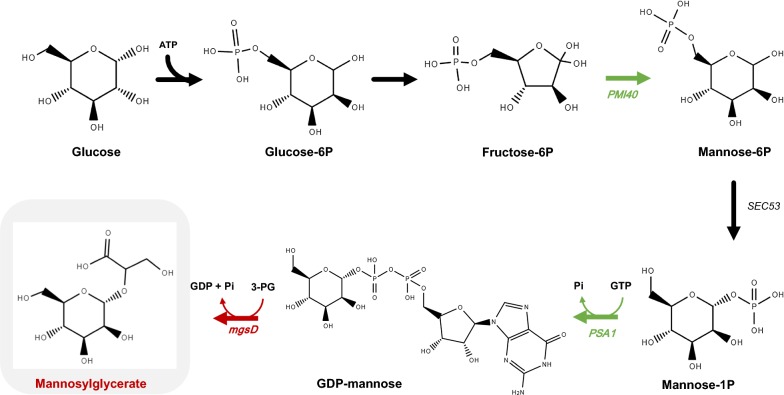
Biosynthesis of mannosylglycerate (MG) in *Saccharomyces cerevisiae* using glucose as carbon source. MG is produced from the reaction of GDP-mannose and 3-phosphoglycerate (3PG) with the release of GMP. To produce MG, a gene from *Dehalococcoides mccartyi* coding for MG synthase/phosphatase (*mgsD*) was cloned in a plasmid and transformed in *S. cerevisiae* to yield strain MG01. A second plasmid containing the genes *PMI40* (encodes a mannose-6-phosphate isomerase) and *PSA1* (encodes a GDP-mannose pyrophosphorylase) from *S. cerevisiae* was constructed and transformed in MG01 yielding MG02

GDP-mannose and 3-phosphoglycerate are the two precursors for the synthesis of MG. We are aware that *S. cerevisiae’s* anabolic pathways imply a high demand for GDP-mannose. Indeed, this activated sugar is the precursor for protein mannosylation and synthesis of oligomannans, important components of the yeast cell wall [23, 24]. Therefore, our first engineering strategy was intended to enhance the flux towards GDP-mannose synthesis by overexpressing the genes encoding mannose-6-phosphate isomerase and GDP-mannose pyrophosphorylase (Fig. 1). The production of MG by the resulting engineered strain was evaluated under different cultivation modes, such as flask bottles, controlled batch and continuous fermentations at different dilution rates.

## Results and discussion

### Strain construction and evaluation of gene overexpression

*Saccharomyces cerevisiae* strain CENPK2-1C was genetically manipulated to overexpress the genes involved in the synthesis of GDP-mannose and MG (Fig. 1). Two engineered strains were constructed: (i) the first strain (named MG01) harbors the plasmid pDES containing the heterologous gene *mgsD* encoding mannosyl-3-phosphoglycerate synthase and mannosyl-3-phosphoglycerate phosphatase; and, (ii) the second strain (named MG02), in addition to plasmid pDES, harbors the plasmid pSP02 containing the mannose-6-phosphate isomerase (*PMI40*) and GDP-mannose pyrophosphorylase (*PSA1*) genes from *S. cerevisiae*. The gene *PMI40* was cloned under the control of *PGK1* promoter and terminator of *CYC1*, while the gene *PSA1* was under the promoter of *TEF1* and the terminator of *ADH1*. These promoters have previously shown high expression levels during growth on glucose [25]. To determine the effect of the overexpression of *PSA1* and *PMI40* genes, mannose-6-phosphate isomerase and GDP-mannose pyrophosphorylase activities were measured in the MG02 strain and compared with the background strain (*S. cerevisiae* strain CENPK2-1C). The MG02 strain showed an increase of twofold in the mannose-6-phosphate isomerase activity and 1.4-fold in the GDP-mannose pyrophosphorylase activity in comparison with the background strain (Table 1).

**Table 1:** Mannose-6-phosphate isomerase (*PMI40*) and GDP-mannose pyrophosphorylase (*PSA1*) activities in the background and MG02 strains

	CENPK2-1C (U/mg of protein)	MG02 (U/mg of protein)	Increase (n-fold)
Mannose-6-phosphate isomerase	120 ± 1	244 ± 10	2.0
GDP-mannose pyrophosphorylase	45 ± 7	65 ± 14	1.4

### Physiological characterization of engineered strains

Cultivation conditions exert a great impact in the performance of producing strains. In particular, *S. cerevisiae* is known to accumulate ethanol as a major by-product in aerobic conditions under certain circumstances, and these different metabolic states may affect the production of the desired compound. When there is a low glucose uptake rate, *S. cerevisiae* exhibits a respiratory metabolism only, with no ethanol formation, while, when the glucose concentration and/or uptake rate surpasses a critical threshold value, the metabolism becomes a combination of respiration and alcoholic fermentation [26]. In this work, the engineered strains were grown under conditions that imposed a respiratory vs respiro-fermentative metabolism; the fitness and MG accumulation parameters were determined under these conditions.

### Physiological characterization of engineered strains in shake flasks and batch bioreactors

The engineered strains were initially characterized in shake flasks using synthetic complete medium (SC medium) with 20 g L^−1^ of glucose. MG01 and MG02 exhibited similar growth rates (0.24 h^−1^ for MG01 vs 0.25 h^−1^ for MG02), but biomass production was different: 0.13 ± 0.01 g_DCW_ g_glc_^−1^ for MG01 and 0.10 ± 0.00 g_DCW_ g_glc_^−1^ for MG02. Profiles of growth, glucose consumption, ethanol, glycerol and acetate production are shown in Additional file 1: Figure S1 indicates a higher production of ethanol and growth for strain MG01 but the same production of glycerol and acetate for both strains. The intracellular content of MG was determined after glucose depletion and, under these conditions, MG02 showed a 1.5-fold increase in MG production compared to the reference strain MG01. A comparison between shake flask and controlled bioreactor for both strains is depicted in Additional file 1: Table S1. *S. cerevisiae* GDP-mannose pool had been successfully increased before by the over-expression of the gene *MPG1* (*PSA1*) in a multi-copy plasmid [23]. In the present work, it was possible to increase MG production by over-expressing *PSA1* (MPG1) and *PMI40,* which re-directed part of the glycolytic flux towards the formation of GDP-mannose and consequently to MG.

**Fig. 2:**
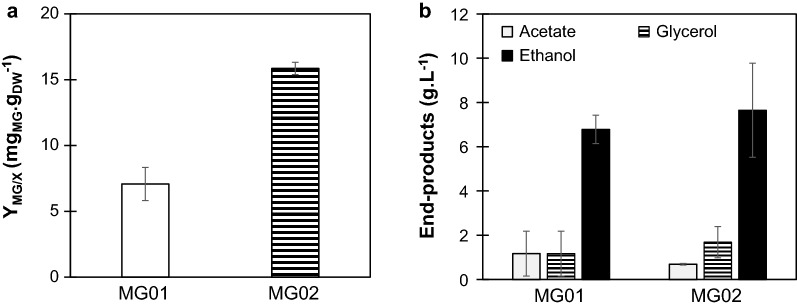
MG production and end-products formation observed for strains MG01 and MG02 in controlled batch cultivations. **a** MG production on biomass for strains MG01 and MG02. Cells were grown in SC medium with 20 g L^−1^ of glucose and MG content was assessed after glucose was depleted from the medium. **b** Ethanol, glycerol, and acetate production of strains MG01 and MG02. Data represents the mean ± SD of three independent experiments

In order to check whether MG accumulation could be enhanced by a controlled environment, strains MG01 and MG02 were grown in a 2-L bioreactor. In controlled batch cultivation, MG02 produced 15.86 mg g_DCW_^−1^ of MG, which represents an increase of 2.2-fold in comparison to MG01 (Fig. 2a). In controlled batch cultivation, using a bioreactor, it is possible to maintain optimal conditions regarding pH, concentration of oxygen and temperature. Under these conditions, strain MG02 produced higher amounts of MG than in shake flasks. Moreover, MG01 and MG02 showed no detectable difference in the production of end-products ethanol, glycerol and acetate (Fig. 2b).

### Physiological characterization of engineered strains in chemostat at different dilution rates

Since the relative contribution of respiratory and respiro-fermentative regimens in *S. cerevisiae* depends on glucose availability, it is difficult to achieve a pure respiratory regimen in controlled batch cultivations. Therefore, a clear distinction of the two metabolic states is only possible in fed-batch and chemostat cultures. In an aerobic environment and in the presence of high concentrations of glucose, *S. cerevisiae* has a fermentative/respiratory metabolism due to a limited respiratory capacity [26]. In addition, there is a molecular response whereby the TCA cycle is repressed and the metabolic flow towards fermentation products is increased, a phenomenon designated as the Crabtree effect [27]. This behavior happens independently of the presence of oxygen to support respiration and is seen as an adaptive response to a competitive environment, as fermentation increases *S. cerevisiae’s* growth rate. Nevertheless, it is possible to ensure a respiratory metabolism as long as the glucose uptake rate is kept below the critical threshold that activates fermentation.

To investigate the impact of the respiratory and fermentative states in MG production, both strains were grown in chemostats at a dilution rate of 0.1 h^−1^, where, in principle, respiration is favored and the production of ethanol is reduced (or even abolished) [28]. Under these conditions, the yield of MG is consistently higher for MG02 in comparison with MG01 (Table 2) and MG productivity is 0.76 mg g_DCW_^−1^ h^−1^ for MG01 and 1.17 mg g_DCW_^−1^ h^−1^ for MG02, which represents a 1.5-fold increase. However, it is apparent that the two strains display different metabolic modes, as MG01 has a Respiratory Quotient (RQ) of 1.39, a value close to fully respiratory metabolism [29], while MG02 has an RQ value of 2.32, which denotes a respiro-fermentative metabolism. This result is also corroborated by the accumulation of higher amounts of ethanol and glycerol in MG02 (Table 2). Despite the RQ differences, the biomass yield under these conditions remains the same for both strains. Most likely, the differences in by-products accumulation are not high enough to provoke an observable decrease in the biomass yield, given the experimental error. Therefore, we conclude that the higher production of MG or/and directly the over-expression of *PSA1* and *PMI40* correlate with a significant decrease of the respiratory capacity of the cell. Although no previous work has reported the physiological impact of overexpressing these two genes, it would be counter-intuitive to assume that a diversion of part of the glycolytic flux would increase by-product accumulation.

**Table 2:** Physiological parameters and MG yield for the engineered strains MG01 and MG02 in chemostat cultivation at dilution rate 0.1 h^−1^

Strain	Y_X/S_^a^	Y_MG/X_^b^	Y_EtOH/S_^a^	Y_Acet/S_^a^	Y_Gly/S_^a^	P_MG/X_^c^
MG01	0.13 ± 0.02	7.58 ± 1.21	0.17 ± 0.03	0.05 ± 0.01	0.001 ± 0.001	0.76 ± 0.12
MG02	0.15 ± 0.02	11.71 ± 0.77	0.33 ± 0.31	0.04 ± 0.02	0.013 ± 0.001	1.17 ± 0.08

^a^Yields on biomass for substrate Y_X/S_ are represented as g_DCW_ g_glc_^−1^ and yields on substrate for ethanol Y_EtOH/S_, acetate Y_Acet/S_ and glycerol Y_Gly/S_ are represented as g g_glc_^−1^

^b^MG production on biomass Y_MG/X_ is represented as mg_MG_ g_DCW_^−1^

^c^Productivity in chemostat cultivation for MG is represented as mg g_DCW_^−1^ h^−1^

Additionally, the ionic nature of MG might be unbalancing cell homeostasis and therefore reducing cell fitness for MG02, as 15.86 mgMG g_DCW_^−1^ represents an intracellular concentration of approximately 25 mM, which is a substantial value for an organism that intrinsically accumulates non-charged osmolytes, like glycerol and trehalose [1, 30]. Increasing concentrations of MG produces an acidification of the cytoplasm (pKa of MG is 3.35) which can compromise growth [31, 32] and impair essential metabolic functions [33]. *S. cerevisiae* cultivated in aerobic conditions with glucose as carbon source, switches from respiratory-fermentative growth to mainly fermentative growth in the presence of high concentrations of acetic acid, that shares with MG the characteristics of a weak acid. Under these conditions, a significant decrease in the specific rate of O_2_ consumption was observed while high level of CO_2_ were maintained [36], as observed for the MG02 strain. Additionally, the metabolic shift to a fermentative behavior (higher RQ) observed in MG02 has been described before for other inhibiting compounds such as furfural, a compound known to inhibit glycolytic enzymes, react with cellular constituents and compromise the respiratory activity [34, 35], although the mechanisms of action are probably different from weak acids.

Additionally, steady-state MG02 cells cultivated under a dilution rate of 0.1 h^−1^ showed a profile of ethanol production that is largely influenced by the concentration of ethanol produced during growth in the batch mode. Chemostat experiments were initiated with different ethanol concentrations, which apparently produced different results for the ethanol concentration at steady-state, explaining the high standard deviation associated with ethanol production observed in Table 2. However, the rate of ethanol decrease from the beginning of the chemostat until steady-state is very similar for both experiments (see Additional file 1: Figure S2). The fact that the initial ethanol concentration is not fully washed from the bioreactor during the continuous cultivation at low dilution rates may be another indication that both strains are not in full respiratory regimen [37]. However, the influence of initial ethanol concentration in continuous cultures in the respiratory capacity has not been, to our knowledge, previously reported and remains unexplained.

To evaluate the impact of increasing the dilution rate, we performed chemostat experiments at dilution rates close to the maximum (washout) values (0.15 h^−1^ for MG02 and 0.2 h^−1^ for MG01). The difference between MG01 and MG02 in MG production observed at 0.1 h^−1^ is kept, while the productivity of both strains in chemostats at 0.1 h^−1^ dilution rate is lower when compared to chemostats performed close to maximal dilution rates (Additional file 1: Table S2). Curiously, MG yield on biomass is not influenced by the variation on the dilution rate, either for MG01 or MG02, although for MG02 the MG yield on substrate is larger for the lower dilution rate.

**Fig. 3:**
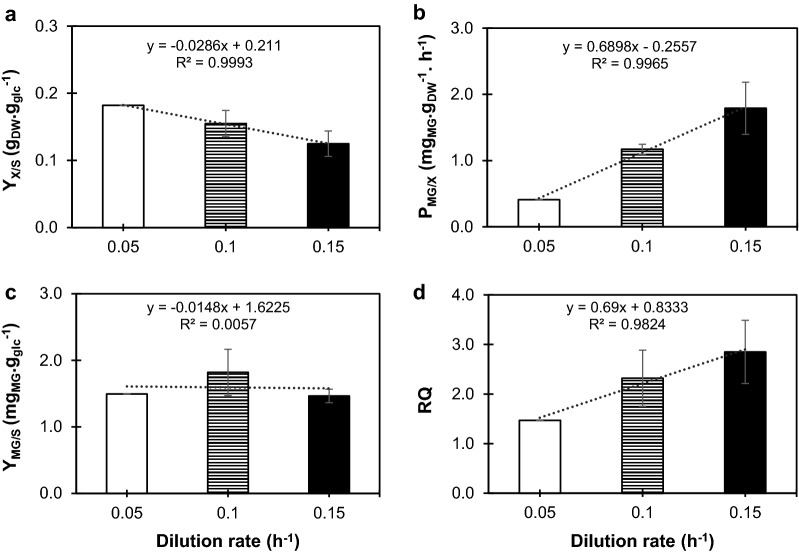
Effect of dilution rate in the formation of biomass, MG production rate and respiratory quotient (RQ) for MG02 (mgsD ↑pmi40 ↑psa1). Chemostats were performed in 2-L fermenters containing SC medium with 20 g L^−1^ glucose. **a** Biomass yield on substrate, represented as g_DCW_ g_glc_^−1^, **b** MG productivity represented as mg_MG_ g_DCW_^−1^ h^−1^, **c** MG yield represented as mg_MG_ g_glc_^−1^, and **d** RQ. For dilution 0.1 and 0.15 h^−1^ data is the mean ± SD of two independent experiments and dilution 0.05 h^−1^ represents one experiment

In order to understand the role of different metabolic states in the production of MG and to gain insight into the respiratory capacity, cells from MG02 were cultivated in a chemostat at dilution rate of 0.05 h^−1^ to allow for fully respiratory mode. A comparison of the three dilution rates for this strain shows that, as expected, the biomass yield on glucose decreases with high dilution rates (Fig. 3a). In aerobic glucose-limited chemostat cultures, it is well-known that when yeast cells are cultivated at high rates (close to the maximal growth rate), the flux from glycolysis is re-directed to the production of by-products (e.g. ethanol) and respiration is kept at lower levels. However, when the dilution rate is reduced, respiration becomes the major source of energy through TCA cycle and consequently, the production of biomass increases [37]. Also, by gradually decreasing the dilution rate in MG02, we verified a decrease in the production rate of MG (Fig. 3b). However, there is no correlation between the dilution rate and MG yield on glucose (Fig. 3c) or even production on biomass (remains the same for dilutions 0.15 and 0.1 h^−1^ (Additional file 1: Table S2) and decreases to 8.21 mg_MG_ g_DCW_^−1^ at dilution 0.05 h^−1^). Despite the lack of correlation with dilution, MG seems to reach a peak of production in terms of yield per substrate at 0.1 h^−1^ dilution rate (Fig. 3c). In light of these results, we can conclude that MG production does not respond linearly to a variation on the growth rate; however, as cells are growing more rapidly, MG productivity is higher at higher dilution rates. Another important result is the variation of RQ values with the dilution rates examined. Coherently with previously published results [29], RQ decreases at lower dilutions and strain MG02 is virtually at a fully respiratory state at dilution 0.05 h^−1^ (Fig. 3d), corroborated by the absence of glycerol production and decrease on acetate/ethanol yields which dropped to 0.1 g g_glc_^−1^.

Continuous mode chemostat presents several advantages in comparison with batch cultures and it is a valuable approach to explore the best conditions for industrial production. One advantage, which is exploited in this work, is the evaluation under controlled conditions of physiological parameters based on growth rates [38]. The cultivation of *S. cerevisiae* in respiro-fermentative metabolic mode holds some issues as it is accompanied by the accumulation of by-products and low biomass yield. Additionally, ethanol production might interfere with the productivity of compounds by provoking toxicity [39]. For this reason, industrial bioprocesses usually use a dilution rate of 0.1 h^−1^ or lower [38] as is the case of resveratrol production [40]. Nonetheless, the respiro-fermentative metabolism favors the production of certain compounds. In a recent study, the cultivation conditions for ethylene production were optimized and were found to be coupled with the growth rate [41]. MG is produced from GDP-mannose and 3-phosphoglycerate (3PG), both derived from glycolysis. As the respiro-fermentative metabolism of *S. cerevisiae* implies a higher glycolytic flux than respiration, it is reasonable to speculate that MG production would increase with higher growth rates in chemostat. Additionally, it has been described that *PSA1* is an essential gene linked to the progression of cell cycle, since efficient glycosylation of proteins is a key-point for cell division [42]. Even more, *PSA1* transcript levels increase with higher growth rates [43], which may favor the synthesis of GDP-mannose and ultimately the synthesis of MG. In the present work, this correlation between MG production and growth rates was not observed. The fact that MG production was not dependent on growth rate may indicate that there is a bottleneck downstream of GDP-mannose, i.e., in the production of MG which is catalyzed by the bifunctional enzyme mannosylglycerate synthase/phosphatase.

## Conclusions

In this study, we have increased the yields and productivity of mannosylgycerate (MG), a protein stabilizer with outstanding properties, by over-expressing *PSA1* and *PMI40* (GDP-mannose pathway) and by optimizing the mode of cultivation. The resulting strain MG02 presents up to 2.2-fold improvement in MG production on biomass, being able to produce 1.79 mg of MG per gram of biomass per hour at a dilution of 0.15 h^−1^. Also, it was not possible to establish a correlation between growth rate and MG yield on substrate, although MG productivity increases with higher dilution rates. New rounds of optimization are needed to produce MG in *S. cerevisiae* at adequate levels, i.e., well above the values reported for organisms that naturally produce this compound. For instance, evaluate the overexpression of SEC53 gene and improve the expression of MG synthase. To this end, a holistic approach should be considered, based on in silico driven metabolic engineering to find gene targets that will improve MG production.

## Methods

### DNA manipulation

*Saccharomyces cerevisiae* genomic DNA for colony PCR and gene amplification was prepared as described by Lõoke et al. [44]. Primers were purchased from Metabion (Germany). Gene amplification and colony PCR were performed using, respectively, Phusion polymerase and DreamTaq DNA polymerase (both enzymes from Thermoscientific, USA). PCR reactions were performed in a Thermal Cycler from Bio-Rad (USA), following the protocols recommended by the manufacturers for each polymerase. Plasmid extraction, PCR product purification, and DNA gel extraction were carried out with Zymo Research kits (USA). All restriction enzymes were obtained in fast-digestion format from Thermoscientific (USA). The plasmid pDES, containing the gene *mgsD* that codes for mannosyl-3-phosphoglycerate synthase/phosphatase from *D. mccartyi* under the control of the *ENO2* promoter from enolase II, was kindly provided by Milton S. da Costa (University of Coimbra, Portugal) [17]. *S. cerevisiae* transformation was performed using the lithium acetate method as described by Gietz et al. [45]. *Escherichia coli* DH5α was used for plasmid isolation and maintenance following the competence and transformation procedures developed by Dower et al. [46].

### Construction of engineered strains

The plasmid pSP-GM was kindly provided by Prof. Jens Nielsen’s group (Chalmers University of Technology, Sweden) [25]. This plasmid was used to express the mannose-6-phosphate isomerase (*PMI40*) and GDP-mannose pyrophosphorylase (*PSA1*) encoding genes from *S. cerevisiae*. The gene *PMI40* was amplified using genomic DNA isolated from *S. cerevisiae* strain CENPK2-1C with the primers pair 5′-CCGCGGCCGCAAAAAAATGTCCAACAAGCTGTTCAGG-3′ and 5′-CCGAGCTCCTAATTTGGTTCCACAAAGGC-3′. This PCR product was digested with *Sac*I/*Not*I and ligated into pSP-GM between promoter of *TEF1* and terminator of *ADH1* using a T4 ligase (Thermoscientific, USA), yielding pSP01. Next, the gene *PSA1* was amplified using again *S. cerevisiae* strain CENPK2-1C genomic DNA with primers pair 5′-GGCCCGGGAAAAAAATGAAAGGTTTAATTTTAGTCGG-3′ and 5′-CCAAGCTTTCACATAATAATAGCTTCCTTTGG-3′. This PCR product was digested with *Hin*dIII/*Xma*I and ligated into pSP01 using a T4 ligase between promoter of *PGK* and terminator of *CYC1*, resulting in plasmid pSP02. Colonies harboring pSP01 and pSP02 were identified by performing colony PCR with the primers used in the amplification. Correct constructions of plasmids were confirmed by restriction analysis and DNA sequencing (GATC Biotech, Germany).

The strain *S. cerevisiae* CENPK2-1C, obtained from EUROCARF (Germany), was used as the host strain in this work. The strain MG01 was obtained by transforming CENPK2-1C cells with plasmid pDES (conferring yeast cells the ability to produce MG); strain MG02 was obtained by transforming MG01 with plasmid pSP02, leading to a strain combining GDP-mannose overproduction and MG synthesis (Table 3).

**Table 3:** List of strains and plasmids used in this work

	Relevant characteristics	References
Strains		
CEN.PK2-1C	Wild-type strain (MATa; his3D1; leu2-3_112; ura3-52; trp1-289; MAL2-8c; SUC2)	[47]
MG01	CEN.PK2-1C with plasmid pDES to allow MG production	This work
MG02	CEN.PK2-1C with plasmid pDES and plasmid pSP02 to overexpress genes from GDP-mannose pathway	This work
Plasmids		
pDES	pRS425 plasmid backbone with *LEU2* as auxotrophic marker. Contains *mgsD* gene from *D. mccartyi* under the control of the promotor *ENO2* from *S. cerevisiae*	[17]
pSP-GM	pESC plasmid backbone with *URA3* as auxotrophic marker. Harbors TEF1 and PGK promoters from *S. cerevisiae*	[25]
pSP01	pSP-GM with *PMI40* gene from *S. cerevisiae*	This work
pSP02	pSP-GM with *PSA1* and *PMI40* genes from *S. cerevisiae*	This work

*LEU2*, leucine auxotrophy; *mgsD*, mannosyl-3-phosphoglycerate synthase/phosphatase gene; ENO2, gene enolase II; *URA3*, uracil auxotrophy; *PMI40*, mannose-6-phosphate isomerase gene; *PSA1*, GDP-mannose pyrophosphorylase gene

### Strain maintenance and cultivation media

Selection and maintenance of plasmids in *E. coli* was performed in LB medium containing 10 g L^−1^ of peptone, 10 g L^−1^ of NaCl, 5 g L^−1^ of yeast extract, and supplemented with 100 mg L^−1^ of ampicillin. The solid LB medium also included 20 g L^−1^ of agar. All cultivations of *E. coli* were carried out at 37 °C and 200 rpm of agitation.

*Saccharomyces cerevisiae* strain CENPK2-1C (MATa ura3-52 his3-Δ1 leu2-3,112 trp1-289, MAL2-8c SUC2) was cultivated in YPD medium, containing 10 g L^−1^ of yeast extract, 20 g L^−1^ of peptone, and 20 g L^−1^ of glucose, at 30 °C and 160 rpm. Recombinant strains were selected on Synthetic complete (SC) medium containing 6.7 g L^−1^ of yeast nitrogen base without amino acids from Difco (USA), 20 g L^−1^ of glucose and 20 g L^−1^ of agar. When necessary, histidine (20 mg L^−1^), tryptophan (20 mg L^−1^), uracil (20 mg L^−1^) and leucine (30 mg L^−1^), were added to cover auxotrophies. All cultivations of *S. cerevisiae* were performed at 30 °C with 160 rpm agitation.

The SC medium used for shake flasks, batch in controlled bioreactors, and continuous cultivation contained 6.7 g L^−1^ of yeast nitrogen base without amino acids from Difco (USA) supplemented with 20 g L^−1^ of glucose. For strain MG01, SC medium was supplemented with histidine (20 mg L^−1^), tryptophan (20 mg L^−1^), and uracil (20 mg L^−1^), while for strain MG02 it was complemented only with histidine and tryptophan, in the same concentrations [48]. For shake flask cultivations, MG01 and MG02 were pre-grown in 50 mL of SC medium at 30 °C and 160 rpm. Then, cells were used to inoculate 500 mL shake flasks containing 100 mL of SC medium with an initial OD_600_ of 0.1. Growth was followed using a spectrophotometer V-560 from Jasco (Japan); cells were harvested at the end of the exponential phase (24 h upon inoculation).

Batch cultivations were performed as following: firstly, cells were grown overnight in shake flasks from a single colony. Each fermenter was inoculated at an initial OD_600_ of 0.1. The batch fermentations were performed in a BIOSTAT-B Plus system (Sartorius, Germany) coupled with a 2-L culture vessel. The operating conditions are the following: the fermentation volume was 0.8 L, the temperature was maintained at 30 °C, the airflow was 1 vvm, the pH was kept at 5.5 controlled by addition of 2 M NaOH, and the dissolved O_2_ was kept above 30% of saturation by feedback control of the stirring speed from 200 rpm up to 600 rpm. Concentrations of O_2_ and CO_2_ in the exhaust gas were monitored by Bluesens gas analyzers (Germany) and RQ represents the ratio between CO_2_ produced and O_2_ consumed during fermentation. Biological samples were collected at the end of the exponential phase, when CO_2_ production dropped drastically.

Continuous cultivations were also carried out in a Sartorius BIOSTAT-B Plus system coupled with a 2-L culture vessel with a constant working volume of 0.8 L, with the same controlled variables and set-points as before. The medium described above was used to feed the bioreactor at dilution rates of 0.05, 0.1, 0.15 and 0.2 h^−1^. The volume was kept at 0.8 L by controlling the level of broth inside the vessel. To guarantee that the culture reached a steady-state mode, at least 5 volumes of medium were allowed to pass through the culture whenever the dilution rate was changed. Samples were collected when O_2_ consumption and CO_2_ production were constant.

### Quantification of MG, biomass and fermentation end products

For each sampling, 50 mL of broth were removed from the culture and centrifuged at 3600×*g* for 10 min to separate cells from the medium. Next, the supernatant was filtered through a membrane filter with a pore size of 0.22 μm into HPLC vials and stored at − 20 °C until further analyzed for glucose, glycerol, acetate, and ethanol. MG is an intracellular compound and was extracted from the cell pellet by adding 5 mL of water, 5 mL of methanol and 10 mL of chloroform. This mixture was vigorously shaken with the help of a vortex and then centrifuged at 12,000×*g* for 30 min at 4 °C. The top part, corresponding to the aqueous phase, was carefully transferred to a new tube and the process of centrifugation was repeated after the addition of 5 mL of water. Once the aqueous phase was separated, samples were evaporated using a Savant™ SPD131DDA SpeedVac (Thermoscientific, USA). Next, the evaporated samples were dissolved in 1 mL of water and transferred to an HPLC vial. Analysis was performed in an HPLC apparatus from Jasco (Japan) model LC-NetII/ADC equipped with UV-2075 Plus and RI-2031 Plus detectors, also from Jasco. The samples were analyzed using an Aminex HPX-87H column from Bio-Rad, which was kept at 50 °C, and a solution of 0.01 M of H_2_SO_4_ was used as the mobile phase with a flow rate of 0.3 mL min^−1^. Quantitative analysis of glucose, MG, glycerol, acetate and ethanol was performed by comparison with a mixture of standards with known concentrations of each metabolite. MG standards were obtained from cell biomass of *Rhodothermus marinus* as previously described [49]. Calibration curves were prepared using the peak areas of the RI detector for glucose, glycerol, acetate and ethanol and of the UV detector at 210 nm for MG. The cell dry weight was determined by filtering 5 mL of culture broth through a 0.22 μm pore Millipore filter and washing once with 5 mL of water. Filters were then dried for 10 min at 150 W in a microwave oven and weighted using an analytical balance.

### Quantification of enzymatic activities

For the determination of mannose-6-phosphate isomerase and GDP-mannose pyrophosphorylase activities, strains WT and MG02 were grown in shake flasks until mid-exponential phase. Cells were harvested by centrifugation and re-suspended in water with a cocktail of protein inhibitors (1×), following manufacturer’s instructions (Roche, USA). Next, cells were broken with three pulses of vortexing (4.0 m s^−1^ for 60 s) using a Fastprep 24 system (MP, USA), keeping cells on ice for 5 min between each pulse. Supernatants were obtained by centrifugation at 13,500×*g* for 5 min and used to determine enzymatic activities. Total protein was determined using the Bradford method [50]. The spectrophotometric methods used to determine the enzymatic activities have been described by Bergmeyer [51]. Briefly, for the mannose-6-phosphate isomerase activity, the assay mixture (final volume of 1 mL) contained: 50 mM Tris–HCl (pH 7), 5 mM mannose-6-phosphate, 8 mM MgCl_2_, 2.8 mM NADP, 0.27 U glucose-6-phosphate dehydrogenase, 0.27 U phosphoglucose isomerase, and 0.25 mg of cell extract. Reactions were started by the addition of mannose-6-phosphate and absorbance was measured at 340 nm for 2 min. For the GDP-mannose pyrophosphorylase activity, the assay mixture (final volume of 1 mL) contained: 50 mM of Tris–HCl (pH 7), 0.5 mM NaF, 10 mM MgCI_2_, 0.1 mM ADP, 1 mM glucose, 1 mM NADP, 2 mM PPi, 5.5 mM GDP-mannose, 1 U each of nucleoside kinase, hexokinase, and glucose-6-P dehydrogenase, and 0.25 mg of cell extract. The reaction was started by the addition of GDP-mannose, and the formation of NADPH was monitored by recording the absorbance at 340 nm for 2 min. One unit corresponds to the conversion of 1 µmol of substrate per minute.

## Additional file

**Additional file 1: Figure S1.** Growth and end-products formation of the strains MG01 (A) and MG02 (B) cultivated in shake flask with SC medium containing 20 g L^−1^ of glucose. Data represent the mean of three independent experiments. Gly—glycerol; Acet—acetate; EtOH—ethanol. **Table S1.** MG and end-products production and yields for the engineered strains MG01 (mgsD) and MG02 (mgsD ↑pmi40 ↑psa1) cultivated in shake flask and batch fermenters. Data represent the mean ± SD of at least three independent experiments. **Table S2.** Physiological parameters and MG yields for the engineered strains MG01 (mgsD) and MG02 (mgsD ↑pmi40 ↑psa1) in chemostat cultivation at different dilution rates. **Figure S2.** Profile of glucose consumption, acetate, glycerol, ethanol and MG production from the beginning until steady-state for strains MG01 (A) and MG02 (B) cultivated in two independent chemostats (experiment 1 and 2) with dilution 0.1 h^−1^.

## Abbreviations

DCW: dry cell weight
OD_600_: optical density measured at 600 nm
LB medium: Luria–Bertani medium
SC medium: synthetic complete medium
SD: standard deviation
MG: mannosylglycerate
3PG: 3-phosphoglycerate
vvm: gas volume flow per unit of liquid volume per minute
RQ: respiratory quotient

## References

[1] Elbein AD (2003). New insights on trehalose: a multifunctional molecule. Glycobiology.

[2] da Costa MS, Santos H, Galinski EA (1998). An overview of the role and diversity of compatible solutes in Bacteria and Archaea. Adv Biochem Eng Biotechnol.

[3] Santos H, da Costa MS (2002). Compatible solutes of organisms that live in hot saline environments. Environ Microbiol.

[4] Lamosa P, Faria TQ, Borges N, Neves C, Santos H, Glansdorff N, Gerday C (2007). The physiological role, biosynthesis, and mode of action of compatible solutes from (hyper)thermophiles. Physiology and biochemistry of extremophiles.

[5] Santos H, Ramos A, da Costa MS. Thermostabilization, osmoprotection, and protection against desiccation of enzymes, cell components, and cells by mannosylglycerate. European patent submitted at 14/03/97 with no 97670002.1 and published in 07/01/98, Bulletin 1998/02 (EP 816509 A2, A3). Applicant: IBET. Licensed to Bitop AG in 2002; 1998.

[6] Ramos A, Raven NDH, Sharp RJ, Bartolucci S, Rossi M, Cannio R (1997). Stabilization of enzymes against thermal stress and freeze-drying by mannosylglycerate. Appl Environ Microbiol..

[7] Shima S, Hérault DA, Berkessel A, Thauer RK (1998). Activation and thermostabilization effects of cyclic 2,3-diphosphoglycerate on enzymes from the hyperthermophilic *Methanopyrus kandleri*. Arch Microbiol.

[8] Lamosa P, Burke A, Peist R, Huber R, Liu MY, Silva G (2000). Thermostabilization of proteins by diglycerol phosphate, a new compatible solute from the hyperthermophile *Archaeoglobus fulgidus*. Appl Environ Microbiol.

[9] Borges N, Ramos A, Raven NDH, Sharp RJ, Santos H (2002). Comparative study of the thermostabilizing properties of mannosylglycerate and other compatible solutes on model enzymes. Extremophiles.

[10] Longo CM, Wei Y, Roberts MF, Miller SJ (2009). Asymmetric syntheses of l, l- and l,d-di-myo-inositol-1,1′-phosphate and their behavior as stabilizers of enzyme activity at extreme temperatures. Angew Chemie Int Ed..

[11] Santos H, Lamosa P, Borges N, Gonçalves LG, Pais T, Rodrigues MV (2011). Organic compatible solutes of prokaryotes that thrive in hot environments: the importance of ionic compounds for thermostabilization.

[12] Jorge CD, Borges N, Bagyan I, Bilstein A, Santos H (2016). Potential applications of stress solutes from extremophiles in protein folding diseases and healthcare. Extremophiles.

[13] Faria TQ, Mingote A, Siopa F, Ventura R, Maycock C, Santos H (2008). Design of new enzyme stabilizers inspired by glycosides of hyperthermophilic microorganisms. Carbohydr Res.

[14] Faria C, Jorge CD, Borges N, Tenreiro S, Outeiro TF, Santos H (2013). Inhibition of formation of α-synuclein inclusions by mannosylglycerate in a yeast model of Parkinson’s disease. Biochim Biophys Acta.

[15] Martins LO, Empadinhas N, Marugg JD, Miguel C, Ferreira C, da Costa MS (1999). Biosynthesis of mannosylglycerate in the thermophilic bacterium *Rhodothermus marinus*. Biochemical and genetic characterization of a mannosylglycerate synthase. J Biol Chem.

[16] Borges N, Jorge CD, Gonçalves LG, Gonçalves S, Matias PM, Santos H (2014). Mannosylglycerate: structural analysis of biosynthesis and evolutionary history. Extremophiles.

[17] Empadinhas N, Albuquerque L, Costa J, Zinder SH, Santos M, Santos H (2004). A gene from the mesophilic bacterium *Dehalococcoides ethenogenes* encodes a novel mannosylglycerate synthase. J Bacteriol.

[18] Asadollahi MA, Maury J, Patil KR, Schalk M, Clark A, Nielsen J (2009). Enhancing sesquiterpene production in *Saccharomyces cerevisiae* through in silico driven metabolic engineering. Metab Eng.

[19] van Zyl WH, Lynd LR, den Haan R, McBride JE (2007). Consolidated bioprocessing for bioethanol production using *Saccharomyces cerevisiae*. Adv Biochem Eng Biotechnol.

[20] Ro D-K, Paradise EM, Ouellet M, Fisher KJ, Newman KL, Ndungu JM (2006). Production of the antimalarial drug precursor artemisinic acid in engineered yeast. Nature.

[21] Otero JM, Cimini D, Patil KR, Poulsen SG, Olsson L, Nielsen J (2013). Industrial systems biology of *Saccharomyces cerevisiae* enables novel succinic acid cell factory. PLoS ONE.

[22] Brochado AR, Matos C, Møller BL, Hansen J, Mortensen UH, Patil KR (2010). Improved vanillin production in baker’s yeast through in silico design. Microb Cell Fact.

[23] Janik A, Sosnowska M, Kruszewska J, Krotkiewski H, Lehle L, Palamarczyk G (2003). Overexpression of GDP-mannose pyrophosphorylase in *Saccharomyces cerevisiae* corrects defects in dolichol-linked saccharide formation and protein glycosylation. Biochim Biophys Acta Gen Subj..

[24] Hashimoto H, Sakakibara A, Yamasaki M, Yoda K (1997). *Saccharomyces cerevisiae* VIG9 encodes GDP-mannose pyrophosphorylase, which is essential for protein glycosylation. J Biol Chem.

[25] Partow S, Siewers V, Bjørn S, Nielsen J, Maury J (2010). Characterization of different promoters for designing a new expression vector in *Saccharomyces cerevisiae*. Yeast.

[26] Sonnleitner B, Käppeli O (1986). Growth of *Saccharomyces cerevisiae* is controlled by its limited respiratory capacity: formulation and verification of a hypothesis. Biotechnol Bioeng.

[27] Crabtree HG (1929). Observations on the carbohydrate metabolism of tumours. Biochem J..

[28] Postma E, Verduyn C, Scheffers WA, Van Dijken JP (1989). Enzymic analysis of the crabtree effect in glucose-limited chemostat cultures of *Saccharomyces cerevisiae*. Appl Environ Microbiol.

[29] Otterstedt K, Larsson C, Bill RM, Ståhlberg A, Boles E, Hohmann S (2004). Switching the mode of metabolism in the yeast *Saccharomyces cerevisiae*. EMBO Rep Eur Mol Biol Organ.

[30] Nevoigt E, Stahl U (1997). Osmoregulation and glycerol metabolism in the yeast *Saccharomyces cerevisiae*. FEMS Microbiol Rev.

[31] Russell JB (1992). Another explanation for the toxicity of fermentation acids at low pH: anion accumulation versus uncoupling. J Appl Bacteriol.

[32] Ullah A, Orij R, Brul S, Smits GJ (2012). Quantitative analysis of the modes of growth inhibition by weak organic acids in *Saccharomyces cerevisiae*. Appl Environ Microbiol.

[33] Abbott DA, Knijnenburg TA, de Poorter LMI, Reinders MJT, Pronk JT, van Maris AJA (2007). Generic and specific transcriptional responses to different weak organic acids in anaerobic chemostat cultures of *Saccharomyces cerevisiae*. FEMS Yeast Res.

[34] Sárvári Horváth I, Franzén CJ, Taherzadeh MJ, Niklasson C, Lidén G (2003). Effects of furfural on the respiratory metabolism of *Saccharomyces cerevisiae* in glucose-limited chemostats. Appl Environ Microbiol.

[35] Heer D, Sauer U (2008). Identification of furfural as a key toxin in lignocellulosic hydrolysates and evolution of a tolerant yeast strain. Microb Biotechnol.

[36] Guo Z, Olsson L (2014). Physiological response of *Saccharomyces cerevisiae* to weak acids present in lignocellulosic hydrolysate. FEMS Yeast Res.

[37] Van Hoek P, Van Dijken JP, Pronk JT (1998). Effect of specific growth rate on fermentative capacity of baker’s yeast. Appl Environ Microbiol.

[38] Nielsen J, Villadsen J, Lidén G (2003). Bioreaction engineering principles.

[39] Stanley D, Bandara A, Fraser S, Chambers PJ, Stanley GA (2010). The ethanol stress response and ethanol tolerance of *Saccharomyces cerevisiae*. J Appl Microbiol.

[40] Vos T, de la Torre Cortés P, van Gulik WM, Pronk JT, Daran-Lapujade P (2015). Growth-rate dependency of de novo resveratrol production in chemostat cultures of an engineered *Saccharomyces cerevisiae* strain. Microb Cell Fact.

[41] Johansson N, Quehl P, Norbeck J, Larsson C (2013). Identification of factors for improved ethylene production via the ethylene forming enzyme in chemostat cultures *of Saccharomyces cerevisiae*. Microb Cell Fact.

[42] Zhang N, Gardner DC, Oliver SG, Stateva LI (1999). Down-regulation of the expression of PKC1 and SRB1/PSA1/VIG9, two genes involved in cell wall integrity in *Saccharomyces cerevisiae*, causes flocculation. Microbiology.

[43] Nookaew I, Papini M, Pornputtapong N, Scalcinati G, Fagerberg L, Uhlén M (2012). A comprehensive comparison of RNA-Seq-based transcriptome analysis from reads to differential gene expression and cross-comparison with microarrays: a case study in *Saccharomyces cerevisiae*. Nucleic Acids Res.

[44] Lõoke M, Kristjuhan K, Kristjuhan A (2011). Extraction of genomic DNA from yeasts for PCR-based applications. Biotechniques.

[45] Gietz RD, Woods RA (2002). Transformation of yeast by lithium acetate/single-stranded carrier DNA/polyethylene glycol method. Methods Enzymol.

[46] Dower WJ, Miller JF, Ragsdale CW (1988). High efficiency transformation of *E. coli* by high voltage electroporation. Nucleic Acids Res.

[47] Entian K-D, Kötter P (2007). 25 yeast genetic strain and plasmid collections. Methods Microbiol.

[48] Guthrie C, Fink GR (1991). Guide to yeast genetics and molecular biology.

[49] Santos H, Lamosa P, Borges N (2006). Characterization and quantification of compatible solutes in (hyper)thermophilic microorganisms. Methods Microbiol..

[50] Bradford MM (1976). A rapid and sensitive method for the quantitation of microgram quantities of protein utilizing the principle of protein-dye binding. Anal Biochem.

[51] Bergmeyer HU, Gawehn K (1974). Methods of enzymatic analysis.

