# Supplementary material for: Production of mannosylglycerate in Saccharomyces cerevisiae by metabolic engineering and bioprocess optimization
Source: Microb Cell Fact. 2018 Nov 16;17:178. doi: 10.1186/s12934-018-1023-7 (PMC6240254; doi:10.1186/s12934-018-1023-7)
Supplement: Supplementary file 1 — Additional file 1: Figure S1. Growth and end-products formation of the strains MG01 (A) and MG02 (B) cultivated in shake flask with SC medium containing 20 g L−1 of glucose. Data represent the mean of three independent experiments. Gly—glycerol; Acet—acetate; EtOH—ethanol. Table S1. MG and end-products production and yields for the engineered strains MG01 (mgsD) and MG02 (mgsD ↑pmi40 ↑psa1) cultivated in shake flask and batch fermenters. Data represent the mean ± SD of at least three independent experiments. Table S2. Physiological parameters and MG yields for the engineered strains MG01 (mgsD) and MG02 (mgsD ↑pmi40 ↑psa1) in chemostat cultivation at different dilution rates. Figure S2. Profile of glucose consumption, acetate, glycerol, ethanol and MG production from the beginning until steady-state for strains MG01 (A) and MG02 (B) cultivated in two independent chemostats (experiment 1 and 2) with dilution 0.1 h−1. [file 12934_2018_1023_MOESM1_ESM.pptx]

## Slide 1
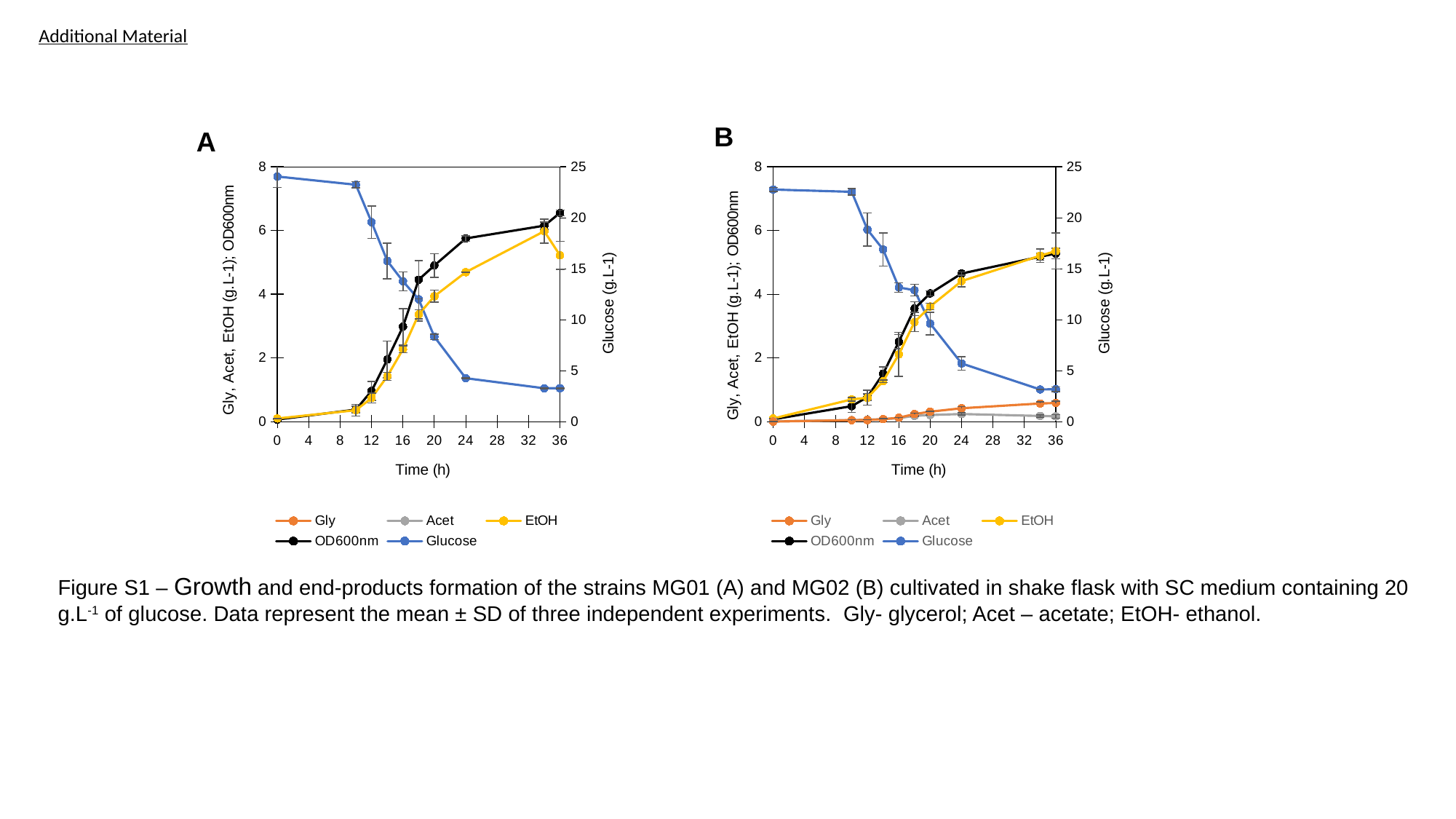

Additional Material
B
A
### Chart
| Category | Gly | Acet | EtOH | OD600nm | Glucose |
|---|---|---|---|---|---|
### Chart
| Category | Gly | Acet | EtOH | OD600nm | Glucose |
|---|---|---|---|---|---|Figure S1 – Growth and end-products formation of the strains MG01 (A) and MG02 (B) cultivated in shake flask with SC medium containing 20 g.L-1 of glucose. Data represent the mean ± SD of three independent experiments. Gly- glycerol; Acet – acetate; EtOH- ethanol.

## Slide 2
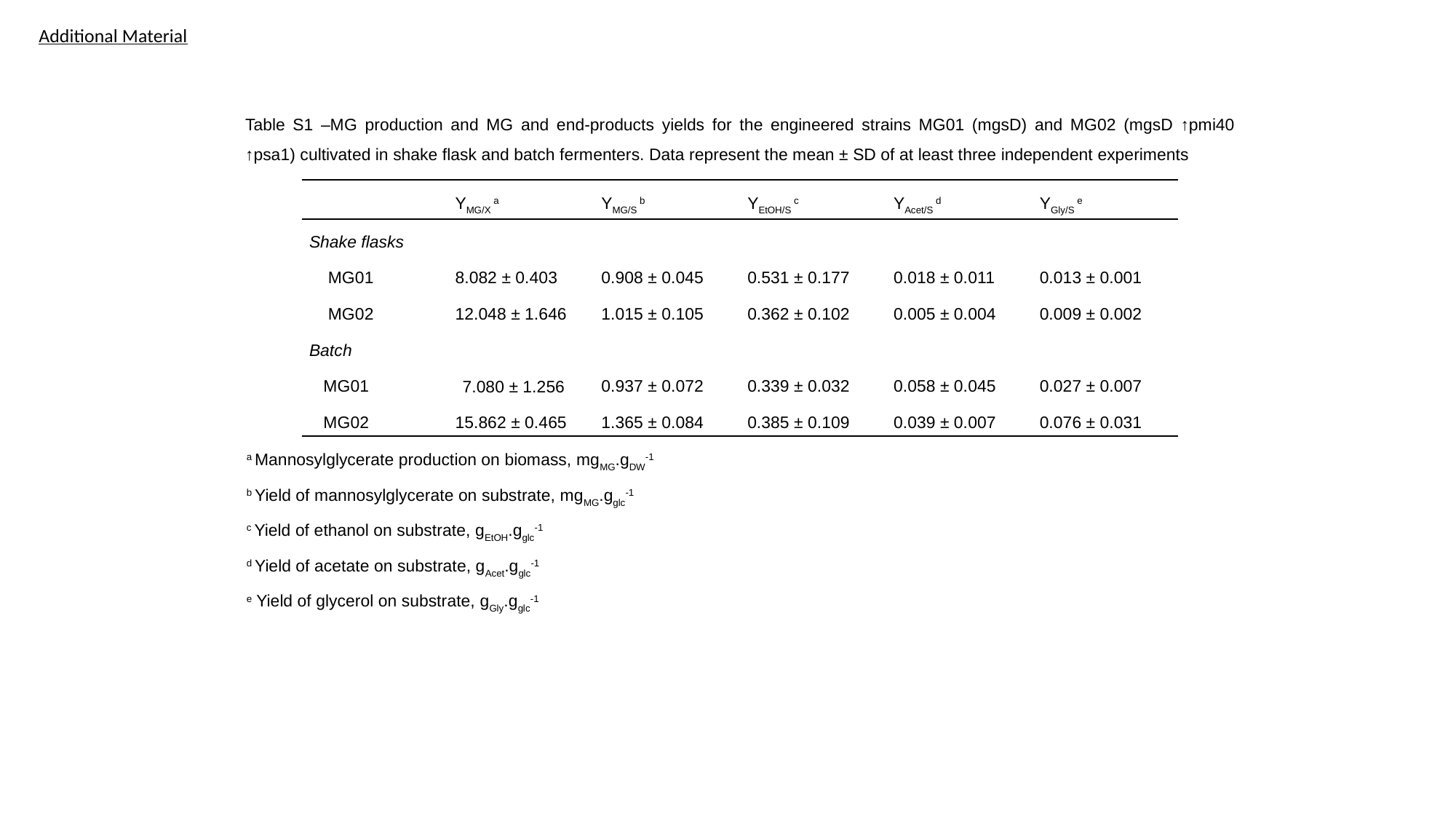

Additional Material
Table S1 –MG production and MG and end-products yields for the engineered strains MG01 (mgsD) and MG02 (mgsD ↑pmi40 ↑psa1) cultivated in shake flask and batch fermenters. Data represent the mean ± SD of at least three independent experiments
| | YMG/X a | YMG/S b | YEtOH/S c | YAcet/S d | YGly/S e |
| --- | --- | --- | --- | --- | --- |
| Shake flasks | | | | | |
| MG01 | 8.082 ± 0.403 | 0.908 ± 0.045 | 0.531 ± 0.177 | 0.018 ± 0.011 | 0.013 ± 0.001 |
| MG02 | 12.048 ± 1.646 | 1.015 ± 0.105 | 0.362 ± 0.102 | 0.005 ± 0.004 | 0.009 ± 0.002 |
| Batch | | | | | |
| MG01 | 7.080 ± 1.256 | 0.937 ± 0.072 | 0.339 ± 0.032 | 0.058 ± 0.045 | 0.027 ± 0.007 |
| MG02 | 15.862 ± 0.465 | 1.365 ± 0.084 | 0.385 ± 0.109 | 0.039 ± 0.007 | 0.076 ± 0.031 |
a Mannosylglycerate production on biomass, mgMG.gDW-1
b Yield of mannosylglycerate on substrate, mgMG.gglc-1
c Yield of ethanol on substrate, gEtOH.gglc-1
d Yield of acetate on substrate, gAcet.gglc-1
e Yield of glycerol on substrate, gGly.gglc-1

## Slide 3
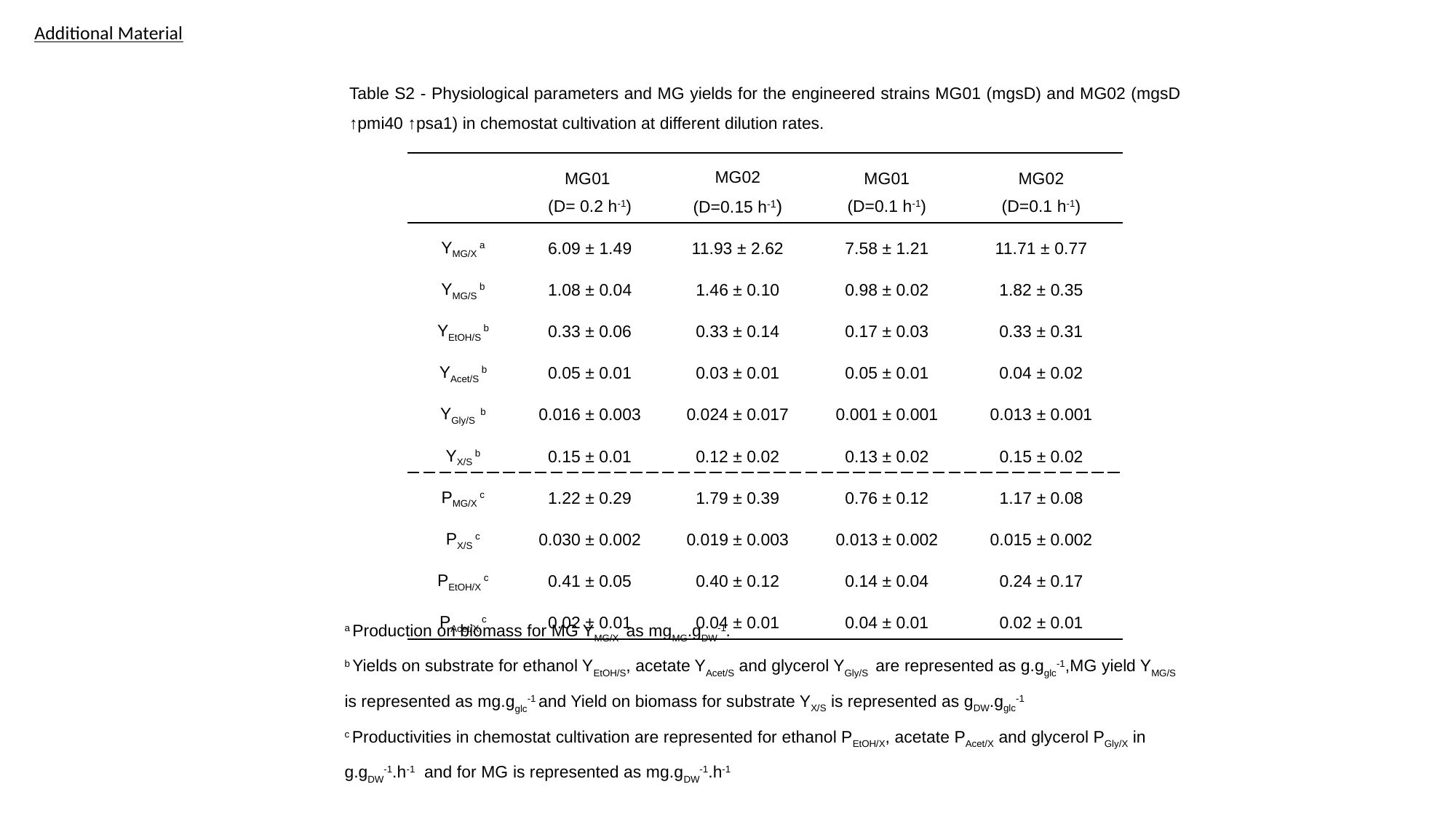

Additional Material
Table S2 - Physiological parameters and MG yields for the engineered strains MG01 (mgsD) and MG02 (mgsD ↑pmi40 ↑psa1) in chemostat cultivation at different dilution rates.
| | MG01 (D= 0.2 h-1) | MG02 (D=0.15 h-1) | MG01 (D=0.1 h-1) | MG02 (D=0.1 h-1) |
| --- | --- | --- | --- | --- |
| YMG/X a | 6.09 ± 1.49 | 11.93 ± 2.62 | 7.58 ± 1.21 | 11.71 ± 0.77 |
| YMG/S b | 1.08 ± 0.04 | 1.46 ± 0.10 | 0.98 ± 0.02 | 1.82 ± 0.35 |
| YEtOH/S b | 0.33 ± 0.06 | 0.33 ± 0.14 | 0.17 ± 0.03 | 0.33 ± 0.31 |
| YAcet/S b | 0.05 ± 0.01 | 0.03 ± 0.01 | 0.05 ± 0.01 | 0.04 ± 0.02 |
| YGly/S b | 0.016 ± 0.003 | 0.024 ± 0.017 | 0.001 ± 0.001 | 0.013 ± 0.001 |
| YX/S b | 0.15 ± 0.01 | 0.12 ± 0.02 | 0.13 ± 0.02 | 0.15 ± 0.02 |
| PMG/X c | 1.22 ± 0.29 | 1.79 ± 0.39 | 0.76 ± 0.12 | 1.17 ± 0.08 |
| PX/S c | 0.030 ± 0.002 | 0.019 ± 0.003 | 0.013 ± 0.002 | 0.015 ± 0.002 |
| PEtOH/X c | 0.41 ± 0.05 | 0.40 ± 0.12 | 0.14 ± 0.04 | 0.24 ± 0.17 |
| PAcet/X c | 0.02 ± 0.01 | 0.04 ± 0.01 | 0.04 ± 0.01 | 0.02 ± 0.01 |
a Production on biomass for MG YMG/X as mgMG.gDW-1.
b Yields on substrate for ethanol YEtOH/S, acetate YAcet/S and glycerol YGly/S are represented as g.gglc-1,MG yield YMG/S is represented as mg.gglc-1 and Yield on biomass for substrate YX/S is represented as gDW.gglc-1
c Productivities in chemostat cultivation are represented for ethanol PEtOH/X, acetate PAcet/X and glycerol PGly/X in g.gDW-1.h-1 and for MG is represented as mg.gDW-1.h-1

## Slide 4
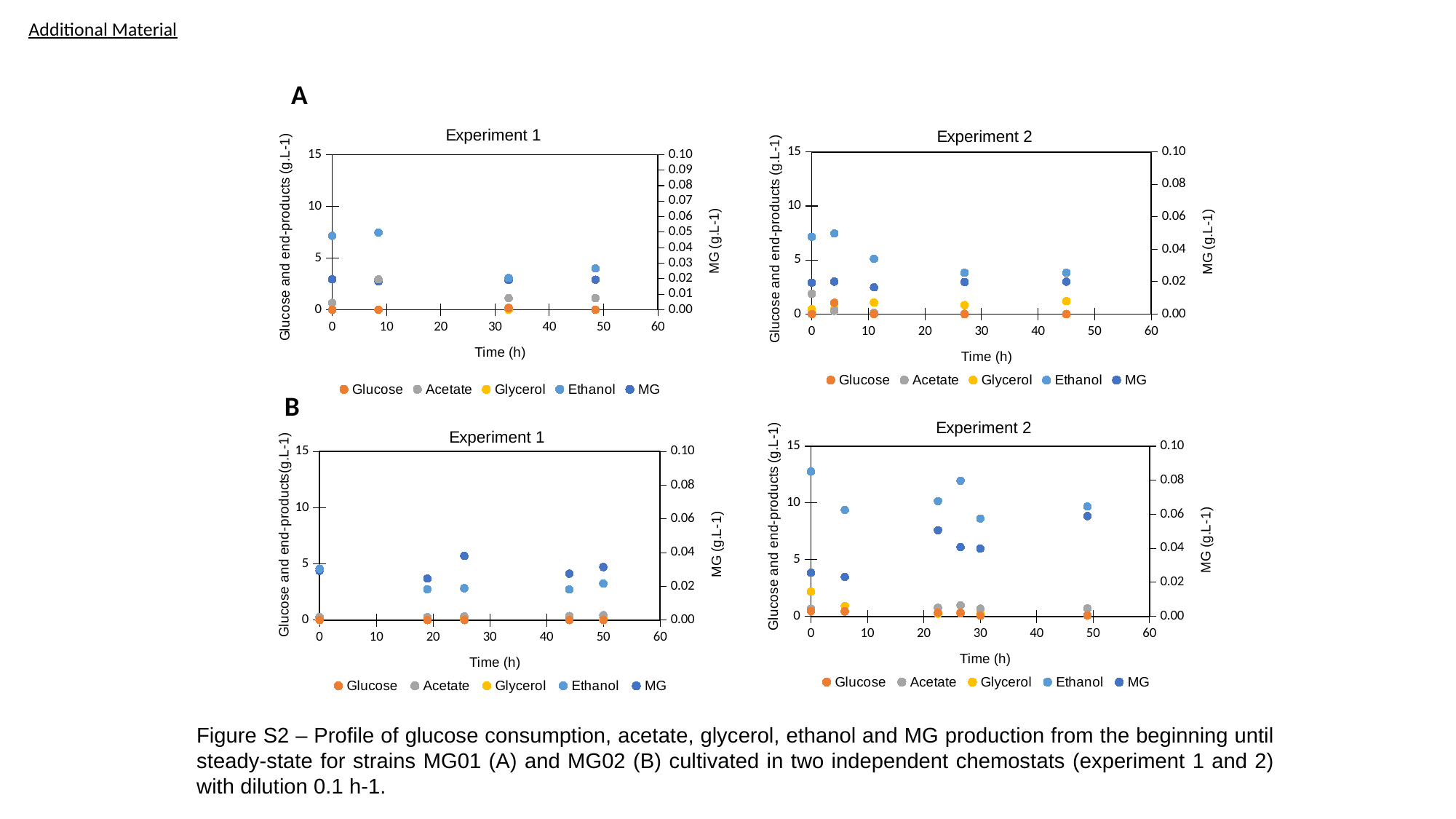

Additional Material
A
### Chart: Experiment 1
| Category | Glucose | Acetate | Glycerol | Ethanol | MG |
|---|---|---|---|---|---|
### Chart: Experiment 2
| Category | Glucose | Acetate | Glycerol | Ethanol | MG |
|---|---|---|---|---|---|B
### Chart: Experiment 2
| Category | Glucose | Acetate | Glycerol | Ethanol | MG |
|---|---|---|---|---|---|
### Chart: Experiment 1
| Category | Glucose | Acetate | Glycerol | Ethanol | MG |
|---|---|---|---|---|---|Figure S2 – Profile of glucose consumption, acetate, glycerol, ethanol and MG production from the beginning until steady-state for strains MG01 (A) and MG02 (B) cultivated in two independent chemostats (experiment 1 and 2) with dilution 0.1 h-1.
